# Pregnancy: An Underutilized Window of Opportunity to Improve Long-term Maternal and Infant Health—An Appeal for Continuous Family Care and Interdisciplinary Communication

**DOI:** 10.3389/fped.2017.00069

**Published:** 2017-04-13

**Authors:** Birgit Arabin, Ahmet A. Baschat

**Affiliations:** ^1^Center for Mother and Child, Philipps University, Marburg, Germany; ^2^Clara Angela Foundation, Witten, Germany; ^3^Center for Fetal Therapy, Johns Hopkins University, Baltimore, MD, USA

**Keywords:** fetal programming, cardiovascular diseases, metabolic diseases, pregnancy as a window for future health, preventive healthcare

## Abstract

Physiologic adaptations during pregnancy unmask a woman’s predisposition to diseases. Complications are increasingly predicted by first-trimester algorithms, amplify a pre-existing maternal phenotype and accelerate risks for chronic diseases in the offspring up to adulthood (Barker hypothesis). Recent evidence suggests that *vice versa*, pregnancy diseases also indicate maternal and even grandparent’s risks for chronic diseases (reverse Barker hypothesis). Pub-Med and Embase were reviewed for Mesh terms “fetal programming” and “pregnancy complications combined with maternal disease” until January 2017. Studies linking pregnancy complications to future cardiovascular, metabolic, and thrombotic risks for mother and offspring were reviewed. Women with a history of miscarriage, fetal growth restriction, preeclampsia, preterm delivery, obesity, excessive gestational weight gain, gestational diabetes, subfertility, and thrombophilia more frequently demonstrate with echocardiographic abnormalities, higher fasting insulin, deviating lipids or clotting factors and show defective endothelial function. Thrombophilia hints to thrombotic risks in later life. Pregnancy abnormalities correlate with future cardiovascular and metabolic complications and earlier mortality. Conversely, women with a normal pregnancy have lower rates of subsequent diseases than the general female population creating the term: “Pregnancy as a window for future health.” Although the placenta works as a gatekeeper, many pregnancy complications may lead to sickness and earlier death in later life when the child becomes an adult. The epigenetic mechanisms and the mismatch between pre- and postnatal life have created the term “fetal origin of adult disease.” Up to now, the impact of cardiovascular, metabolic, or thrombotic risk profiles has been investigated separately for mother and child. In this manuscript, we strive to illustrate the consequences for both, fetus and mother within a cohesive perspective and thus try to demonstrate the complex interrelationship of genetics and epigenetics for long-term health of societies and future generations. Maternal–fetal medicine specialists should have a key role in the prevention of non-communicable diseases by implementing a framework for patient consultation and interdisciplinary networks. Health-care providers and policy makers should increasingly invest in a stratified primary prevention and follow-up to reduce the increasing number of manifest cardiovascular and metabolic diseases and to prevent waste of health-care resources.

## Introduction

**Figure 1:**
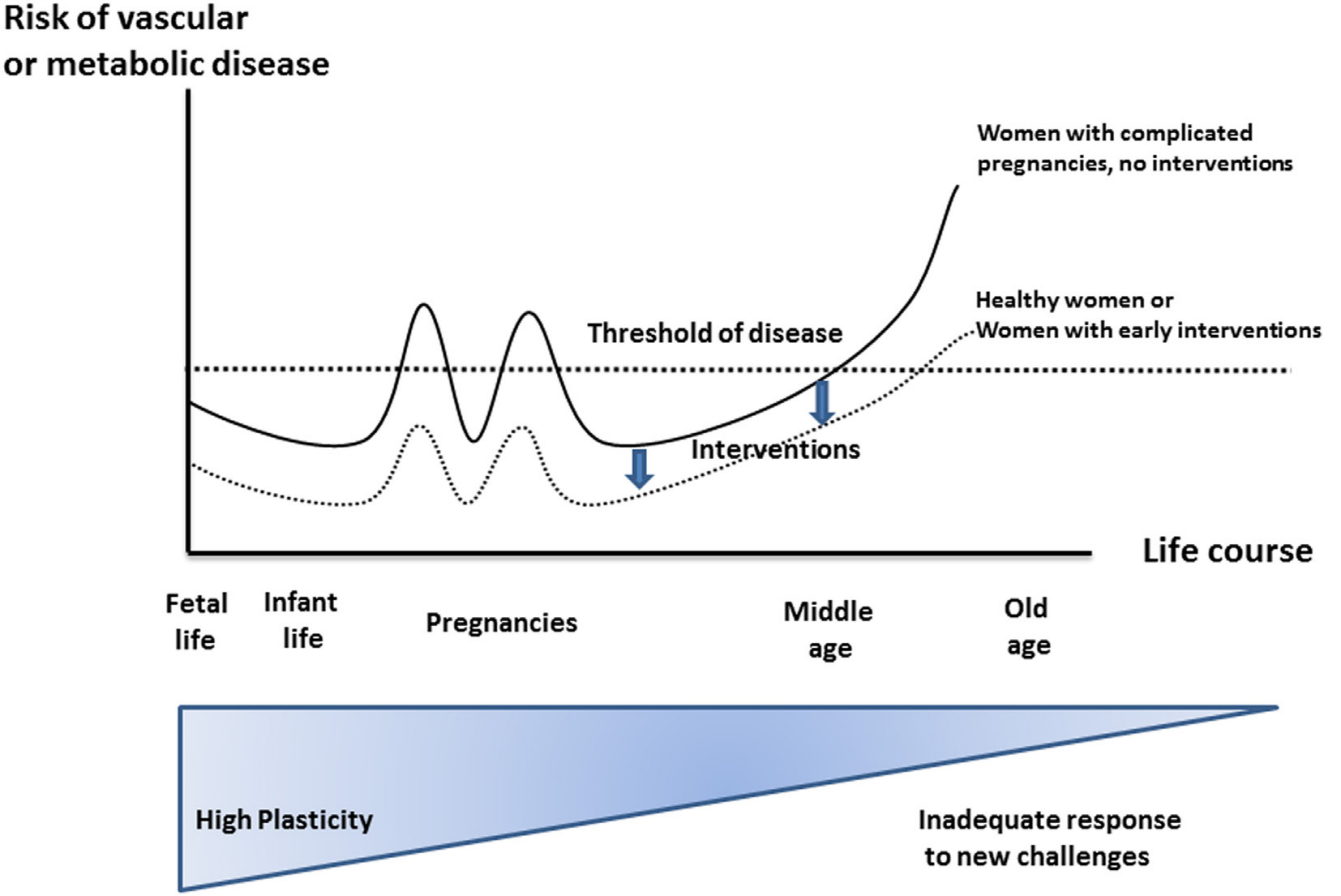
**Effect of developmental environment on later maternal phenotype and pregnancy complications**. Risks for non-communicable diseases increase throughout a life course due to reduced plasticity. Increased risk factors, suspicious first-trimester screening, and pregnancy complications may be considered as the first “markers” and thus allow earlier intervention strategies compared to first symptoms of manifest chronic disease. Modified according to Sattar and Greer (4), Godfrey and Barker (11), and Godfrey et al. (12), similarly designed for Ref. (13).

The care of pregnant women is typically focused on the current pregnancy. Yet, it has been demonstrated that pregnancy complications have lifelong health implications for women and children. Barker et al. correlated low birthweight (LBW) in hunger episodes during World War II with cardiovascular disease (CVD) and type 2 diabetes (1). Several investigators have subsequently confirmed that maternal complications such as preeclampsia (PE), stress, excessive weight gain and gestational diabetes mellitus (GDM) increase chronic disease, and mortality rates in following generations (2, 3). These health risks have been attributed to perinatal programming, a mismatch between prenatally acquired attributes and critical periods in development producing health effects that are independent of a person’s genetic code (Barker hypothesis, Dörners concept of functional teratology). More recently, it was recognized that adverse pregnancy outcome relating to placental syndromes is associated with maternal cardiovascular, metabolic, or thromboembolic risks and earlier mortality (Figure 1) (4). First-trimester screening algorithms now allow individualized prediction of fetal growth restriction (FGR), PE, spontaneous preterm birth (SPB), or GDM (5–8) by utilizing markers of pre-existing maternal “risk-profiles” that not only confer pregnancy-related risks but also lower the thresholds for disease in later life (4, 9, 10).

These observations support the hypothesis that pregnancy can interact with maternal phenotypes and modify risks for “non-communicable diseases” (NCDs) in mothers and children. This is the basis for developing interdisciplinary care paths between obstetricians, general practitioners, internists, and pediatricians that extend beyond the current pregnancy to offer population-based prevention, screening, and individualized secondary prevention. Given the increasing rate of NCDs in low-, middle-, and high-income countries such an approach requires urgent consideration by health-care providers and policy makers.

It is our aim to illustrate how pregnancy itself might serve as a screening tool for future health risks, opening important opportunities for the prevention of the most relevant diseases of our time.

## Methods

Literature searches were performed utilizing Medline, Embase, Science Direct, and Cochrane Library, proceedings from congresses on fetal origins of adult disease, the World Congress of Diabetes in Pregnancy, and the interdisciplinary workshops on the role of pregnancy complications for future maternal and child health hosted by the National Institute of Child Health and Human Development, the Society for Maternal-Fetal medicine, and the American College of Obstetricians and Gynecologists.

We selected studies relating pregnancy risk profiles to long-term cardiovascular and metabolic health of mothers and infants up to adulthood. Search terms included risk profile, miscarriage, PE, FGR, preterm birth, smoking and stress during pregnancy, subfertility, GDM, overweight, obesity and excessive pregnancy weight gain, breastfeeding, and as outcome parameters long-term maternal and infant health, CVD and CVD mortality of mothers and infants, diabetes mellitus (DM) of both mothers and infants, metabolic syndrome, thrombophilia, fetal programming, fetal origin of adult disease, growth, or body composition variable of interest such as fat mass or obesity. Full-text articles were obtained and reviewed to identify those representing the intended context; data were extracted from relevant publications. Results from prospective and retrospective human and animal studies were considered.

There is a significant overlap between cardiovascular, metabolic, and thrombotic profiles in women at risk for placental disease. Prothrombotic risks are usually managed by disease-specific interventions (i.e., anticoagulation for thrombophilia). Accordingly, for the purpose of this review, we have focused on cardiovascular and metabolic risk profiles as major precursors to NCDs to illustrate our concept.

## Results

### Health Risks Attributable to a Cardiovascular Risk Profile

Cardiovascular diseases (heart disease, stroke) account for 31% of all deaths in the United States (US) (14). Over 2,150 Americans/day die of CVD with increasing rates for women aged 35–44 years (14, 15). Higher blood pressures in early adulthood predate increased mortality from all causes (16) and up to 77% of people have blood pressures >140/90 mmHg when they experience a first heart attack or stroke (14). At age 50, total life expectancy for normotensive compared to hypertensive men and women is 5.1, respectively, 4.9 years longer (14). In the last 15 years, the actual number of deaths attributable to high BP rose by 39% whereas the actual number of CVD deaths declined by 15% (14). However, the age-related decrease in mortality was less pronounced in women than in men (17). A mathematical model calculated that a 10% increase of treatment of early hypertension would prevent 14,000 deaths in the US/year (18). In view of these statistics, it is critical to identify those pregnancy complications that lead to persistence of high BP, CVD, and stroke and to consider preventive strategies.

Thilaganathan summarized common and unique characteristics of gestational diabetes and hypertensive disease during pregnancy raising the point that the placenta may not be the initiator of PE. Instead, pre-existing maternal hemodynamics or metabolic diseases may be the primary cause of secondary placental morphology and function (Table 1) (19, 20). While we separate single pregnancy complications for the purpose of this review they may all occur in one woman. In those cases, later health risks for both mother and offspring may exponentially increase.

**Table 1:** **Schematic similarities and differences of gestational diabetes and pregnancy hypertension, with gratitude, according to the work of Thilaganathan (19)**.

	Gestational diabetes mellitus (GDM)	Pregnancy hypertension
**Epidemiology**		
Predisposing factors	Same as for type 2 diabetes	Same as for cardiac disease
Onset of disorder	Mid to late pregnancy	Mid to late pregnancy
Effect of parity	More common in primiparity	More common in primiparity
Recurrence risk	Increased risk if previously affected pregnancy	Increased risk if previously affected pregnancy
**Fetal and placental effects**		
Placental histology	Some histological lesions seen more often in GDM	Some histological lesions seen more often in pregnancy hypertension
Specificity of histology	None of the placental histological lesions specific for the disorder	None of the placental histological lesions specific for the disorder
Temporal nature of lesions	Seen more frequently in early-onset and/or severe disorder	Seen more frequently in early-onset and/or severe disorder
Placental function	Increased maternal-to-fetal transplacental glucose transfer	Impaired maternal perfusion of the uteroplacental bed
Fetus	Increased fetal glucose levels lead to macrosomia	Impaired placental function leads to impaired fetal growth
**Screening/diagnostic tests**		
Mechanism of screening	GTT gauges pancreatic reserve	Uterine Doppler, PIGF and BP are all measures of cardiac function
Performance of screening	Better for early-onset GDM	Better for early-onset preeclampsia (PE)
Timing of screening test	Improved sensitivity the later in pregnancy it is performed	Improved sensitivity the later in pregnancy it is performed
Diagnostic test	Supra-normal glucose levels in both pregnant and non-pregnant	High BP in both pregnant population
**Management**		
Cure for disorder	Birth	Birth
Treatment/amelioration	Insulin—treats the biological deficit	Antihypertensive medications—treat a symptom of the disorder
Long-term maternal health	50% develop type 2 diabetes by 10 years postpartum	20% develop chronic hypertension by 10 years postpartum
**Biology**		
Maternal adaption	Insulin requirements increase twofold to threefold in pregnancy	Cardiac output increases by about 50% in pregnancy
Early-onset phenotypes	Present with normal or lower insulin levels compared to non-pregnancy	Present with normal or lower cardiac outputs compared to non-pregnancy
Late-onset phenotypes	Present with supra-normal (high) insulin levels compared to non-pregnancy	Present with supra-normal (high) cardiac output compared to non-pregnancy
Etiology	Inability of maternal pancreas to deal with the glucose load of pregnancy	Impaired trophoblast invasion or maternal cardiac maladaptation?

#### Miscarriage

A systematic review on effects of early menopause reported that women with early-pregnancy miscarriage are more likely to develop CVD, specifically ischemic heart disease (IHD) in later life, implying lower protective effects of estrogens on serum lipids, and vessel wall anatomy as possible causes (21). Increased total serum cholesterol (22), triglycerides (TGs) (23), antithrombin III (24), factor VII, and fibrinogen (25) have also been speculated to be responsible (22, 26). Early miscarriage, either as a single or recurrent complication, carries a hazard ratio (HR) between 1.25 and 1.56 for subsequent IHD (27) (Table 2). This association was independent of maternal age, height, socioeconomic deprivation, chronic hypertension, and complications during the first pregnancy. The proportion of smokers was only marginally higher compared to women without miscarriage (28.4 vs. 26.8%). While the authors hypothesized that the association of IHD and miscarriage reflects common inherited thrombophilic defects, they did not find significant associations between miscarriage and family history of CVD or venous thrombotic embolism.

**Table 2:** **Impact of pregnancy complications on maternal risks for disease categories in later life (prospective, mostly retrospective cohorts, and systematic reviews)**.

Pregnancy disease	Sample (*n*)	Literature (Reference number/year)	Definition of health risk in later maternal life	Association of health risk HR/OR/RR (95% CI)
**Cardiovascular**				
>1 miscarriage	129,200	(27)/2003	Ischemic heart disease (IHD)	HR 1.52 (1.13–2.06)
2 miscarriages	129,200	(27)/2003	IHD	HR 1.25 (1.04–1.49)
≥3 miscarriages prior to first birth	129,200	(27)/2003	IHD	HR 1.56 (1.14–2.15)
Child with FGR (general)	923,586	(28)/2011	CAD, CVD, cerebrovascular disease	HR 1.39 (1.22–1.58)
Term FGR	923,586	(28)/2011	CAD, CVD, cerebrovascular disease	HR 1.38 (1.15–1.65)
Preterm FGR	923,586	(28)/2011	CAD, CVD, cerebrovascular disease	HR 3.4 (2.26–5.11)
Birthweight	783,814	(29)/2010	Maternal CVD mortality	0.74/kg (0.56–0.99)—inverse relationship
Birthweight	783,814	(29)/2010	Maternal grandfather CVD mortality	1.05 (1.01–1.09)
Birthweight (inverse relationship)	783,814	(29)/2010	Paternal Grandmother CVD mortality	0.93/kg (0.85–1.00)—inverse relationship
Low birthweight <2,500 g	119,668	(30)/2004	Cerebrovascular disease	aHR 2.51 (1.71–3.70)
Multiparity	2,533	(31)/1993	CVD	RR 1.5 (1.1–1.9)
1 birth	1,332,062	(31)/1993	CVD	1.09 (95% CI 1.03–1.15)
>5 births	1,332,062	(31)/1993	CVD	1.47 (95% CI 1.37–1.57)
>2 children	4,286	(32)/2003	Maternal CVD	OR 1.30 (1.17–1.44)
>2 children	4,252	(32)/2003	Paternal CVD	OR 1.12 (1.02–1.22)
Preeclampsia (general)	1,985	(33)/2016	Death at coronary revascularization	HR 1.61 (1.00–2.58)
Maternal placental disease	1,130,764	(34)/2012	Premature heart failure or dysrhythmia	HR 1.51 (1.26–1.80)
Maternal placental disease + FGR	1,130,764	(34)/2012	Premature heart failure or dysrhythmia	HR 2.42 (1.25–4.67)
Maternal placental syndrome	1,030,000	(35)/2005	CVD	HR 2.0 (1.7–2.2)
Maternal placental syndrome + FGR	1,030,000	(35)/2005	CVD	HR 3.1, 2.2–4.5
Maternal placental syndrome + FD	1,030,000	(35)/2005	CVD	HR 4.4, 2.4–7.9
Preterm birth 32–37 weeks	923,686	(28)/2011	CVD	HR 1.39 (1.22–1.58)
Preterm birth 28–31 weeks	923,686	(28)/2011	CVD	HR 2.57 (1.97–3.34)
Spontaneous preterm birth (SPB)	750,350	(36)/2015	Death from IHD	HR 2.26 (1.88–2.71)
SPB	750,350	(36)/2015	Total IDH	HR 1.58 (1.47–1.71)
Preterm birth 32–36 weeks	782,287	(36)/2015	Thromboembolism	aOR 1.42 (1.24–1.62)
**Metabolic**				
(No) Breastfeeding	23,701	(37)/2014	Increased maternal weight after 7 years	β = 0.003 (0.01, 0.003) path analysis, inverse relationship
Breastfeeding (with formula) in patients with GDM	1,010	(38)/2015	Incidence of type 2 DM after 2 years	aHR 0.64 *p* trend = 0.016 (formula = 1)
Breastfeeding (mostly) in patients with GDM	1,010	(38)/2015	Incidence of type 2 DM after 2 years	aHR 0.54 *p* trend = 0.016
Breastfeeding (exclusive) in patients with GDM	1,010	(38)/2015	Incidence of type 2 DM after 2 years	aHR 0.46 *p* trend = 0.016
Pregnancy weight gain > IOM limits	65,000	(39)/2011	3 years postpartum weight gain	3.06 (1.50–4.63) kg, *p* < 0.001
Pregnancy weight gain > IOL limits	65,000	(39)/2011	15 years postpartum weight gain	Mean increase of 4.72 (2.94–6.50) kg
Gestational diabetes	675,455	(40)/2009	Manifest type 2 DM	RR 7.43 (4.79–11.51)
Maternal obesity	46,688	(41)/2016	Hospitalization for CV events	HR 2.6 (2.0–3.4)
Pre-pregnancy BMI > 30 kg/m^2^				
Premature ovarian insufficiency	190,588	(42)/2016	IHD	HR 1.69 (1.29–2.21)
Premature ovarian insufficiency	190,588	(42)/2016	Total CVD	HR 1.61 (1.22–2.12)

*a, adjusted; BMI, body mass index; CAD, coronary artery disease; CVD, cardiovascular disease; DM, diabetes mellitus; FGR, fetal growth restriction; FD, fetal death; HR, hazard ratio; OR, odds ratio; RR, risk ratio (or relative risk)*.

#### Fetal Growth Restriction

It has been long recognized that infant BW correlates with the mother’s subsequent risk for IHD and is “aggregated within families” possibly due to genetic, physiologic, environmental, epigenetic, and socioeconomic factors (30, 43–45). Delivering an FGR infant is associated with maternal coronary artery disease, cerebrovascular disease, or cardiac insufficiency with HRs ranging from 1.35 to 3.4 for severe early-onset FGR (Table 2) (28). Birth weight has an inverse linear association with maternal CVD mortality and is also related to grandparental CVD and CVD mortality (29). These BW associations across generations are independent of socioeconomic, environmental, or behavioral factors, body mass index (BMI), age, or smoking status. Familial aggregation of shared determinants for risk factors associated with pregnancy complications and CVD were postulated as a potential cause for these associations (46). Other hypothesized mechanisms are based on the observation that the impact of the mother is stronger than of the father: women at high risk of CVD may be unable to mount an adequate hemodynamic response leading to FGR and/or PE. This is supported by cardiac changes seen on maternal echocardiography showing increased cardiac chamber dimensions and left ventricular hypertrophy in the index pregnancy. The likelihood of developing FGR and/or PE is elevated by many maternal demographic and medical characteristics, such as hypertension, obesity, and age that are shared risk factors for CVD (47).

Adverse effects of FGR on cardiovascular health of the offspring were already suspected in 1977 by Forsdhal, who reported that Norwegian children, raised in poor provinces in the early twentieth century but became prosperous thereafter, suffered from excess rates of myocardial infarction as adults (48). In 1986, Barker and Osmond showed that the distribution of CVD in England was related to a person’s BW (49). Maternal hunger throughout pregnancy was linked to high blood pressure in the offspring; while third-trimester deprivation led to high levels of low-density lipoprotein, cholesterol, and fibrinogen (50). The Dutch Famine Birth Cohort Study considered long-term effects of prenatal starvation among women born before, during, or after the Hunger Winter when the average supply was <1,000 calories/day (51). Intra-family sibling analysis revealed that BW was decreased when famine exposure was in the third trimester but not when it was in the first trimester. However, the expected increase in BW of the offspring with birth order was reversed after maternal exposure in the first trimester (52). The results suggested that biologic effects depend on timing of gestational exposure and are still present in subsequent generations. People conceived during the famine had doubled rates of CVD, atherogenic plasma lipid profiles and were at increased risk of schizophrenia, depression, high stress responsiveness and performed worse on tasks that correlated with accelerated aging (53) (Table 3).

**Table 3:** **Impact of pregnancy complications on fetal risks for disease categories up to adulthood (selected prospective, mostly retrospective cohorts, and systematic reviews)**.

Pregnancy disease	Sample (*n*)	Literature (Reference number/year)	Definition of health risk in fetal life as an adult	Association of health risk HR/OR/relative risk (RR)/SD (95% CI)
**Cardiovascular**				
LBW and famine during gestation	975	(53)/2011	Coronary artery disease	HR 1.9 (1.0 to 3.8) adjusted for sex
LBW and first-trimester famine	726	(53)/2011	CHD	OR 3.0 (1.1 to 8.1) age and sex adjusted
LBW and famine	658	(53)/2011	Systolic BP	−4.14 mmHg/kg (−7.24 to −1.03) inverse relation
			Diastolic BP blood pressure	−2.09 mmHg/kg (−3.77 to −0.41) inverse relation
			Prevalence of hypertension	OR 0.67/kg (−0.49 to 0.93) age and sex adjusted
LBW (SGA) up to HBW (LGA)	6,239	(54)/2016	Left ventricular mass	SD score 0.05 (0.03 to 0.08) *p* < 0.01 for trend
LBW (SGA) up to HBW (LGA)	6,239	(54)/2016	Aortic root diameter	SD score 0.08 (0.05 to 0.1) *p* < 0.01 for trend
Preeclampsia (PE)	45,249	(55)/2013	High systolic BP during child and adulthood	2.39 mmHg (1.74 to 3.05)
			High diastolic BP during child and adulthood	1.35 mmHg (0.90 to 1.80)
PE	2,868	(56)/2015	Cardiovascular risk, hypertension and metabolic disease (QRISK > 75 P) at age 20	OR 2.5 (1.32 to 4.56)
		Prospective cohort		
Complicated HTN + birth factors	2,868	(56)/2015	Hypertension at age 20	aOR 6.25 (1.96 to 19.96)
Complicated HTN + risks at 20 years				aOR 6.74 (1.25 to 36.29)
Complicated HTN + social risks				aOR 6.63 (1.17 to 37.57)
Preterm SGA vs. term	1,756	(83)/2015	Adult hypertension	36.9 vs. 25.4%; risk factors adjusted *p* = 0.006
**Neurologic**				
First-trimester famine	66,321	(53)/2011	Schizophrenia ICD-6/-9	OR 2.01 (1.03 to 3.94)
First-trimester famine	737	(53)/2011	Accelerated aging, depression	β = −85 (−139 to −32), *p* = 0.002
First- and second-trimester famine	100,543	(53)/2011	Antisocial personality disorder in men ICD-6	aOR 2.5 (1.5 to 4.2)
Fetal growth restriction	1,679	(57)/2008	Hostility	(beta)SD < −0.05 (−0.14 to 0.00)
**Metabolic**				
First-trimester famine	2,414	(53)/2011	Increase LDL–high-density lipoprotein cholesterol ratios adult	Increase 13.9% (2.6 to 26.4)
LBW	2,546	(58)/2016	Leptin to fat mass ratio, leptin, diabetes mellitus (DM), obesity	*p* < 0.05 for all, chi-square test or ANOVA
Complicated HTN + birth factors	2,868	(56)/2015	Overweight or obesity at age 20	aOR 1.68 (1.18 to 2.39)
Complicated HTN + risks at 20 years		Prospective cohort		aOR 1.62 (1.05 to 2.52)
Complicated HTN + social risks				aOR 1.59 (1.02 to 2.48)
Early preterm	1,358	(59)/1996	High insulin at birth	OR 2.05 (1.69 to 2.42)
Early preterm	1,358	(59)/1996	High insulin in childhood	OR 1.31 (1.10 to 1.52)
Parental smoking	17,003	(60)/2014	Increased body mass index (BMI) and WC at 32 years	Increase of 0.57 kg/m^2^/1.46 cm (*p* ≤ 0.02)
Birth weight < 2 SD	61,311	(61)/2007	DM as an adult	OR 2.01 (1.39 to 2.91)
Birth weight ≥ 2 SD			DM as an adult	OR 2.27 (1.38 to 3.74)
Famine second trimester	702	(62)/2006	Decreased glucose tolerance at 50/58 years	Diff = 0.4 mmol/l (0.1 to 0.7), sex/BMI adjusted
LGA and GDM during pregnancy	179	(63)/2005	Metabolic syndrome at 11 years (increased insulin resistance and obesity)	OR 10.4 (1.5 to 74.4)
After ovulation induction	2,577	(64)/2014	Increased fasting glucose levels at 6 years	0.4 mmol/l (0.2 to 0.6)
After *in vitro* fertilization	2,577	(64)/2014	Increased fasting glucose levels at 6 years	0.2 mmol/l (0.0 to 0.5)

*a, adjusted; BP, blood pressure; CHD, coronary heart disease; GDM, gestational diabetes mellitus; HR, hazard ratio; HTN, hypertensive disease in pregnancy; LBW, low birthweight; LGA, large for gestational age; OR, odds ratio; SGA, small for gestational age*.

In most pregnancy studies, the effects of famine cannot be clearly separated from those of stress especially when studied in the context of war trauma. Following the Chernobyl disaster, the perceived level of stress was a better predictor of the offspring’s risk of cognitive disorders than the actual exposure to radiation (65). Conditions during and after famine periods varied between countries: in the Netherlands, World War II was followed by a period of abundance and in Russia, life conditions remained poor. The latter was not associated with adverse effects suggesting that it is beneficial when the postnatal environment matches the prenatal environment (66). There are also individual differences in susceptibility toward hunger and stress dependent on the individual genetic background (67). While starvation may play a minor role in high resourced countries, the first trimester is a vulnerable period as serious hyperemesis significantly increases the odds for cognitive and psychological diseases in the offspring (OR 3.6, *p* < 0.0001) (68).

Cardiovascular changes in FGR offspring are already present *in utero*, can persist after birth, and may produce aberrant cardiomyocyte growth in adult hearts with reversible myocardial hypertrophy (69). In humans, FGR induced cardiovascular remodeling at age 5 is ameliorated by breastfeeding but worsened by a high maternal BMI (70). Longitudinal growth analysis suggests that children with higher BP tended to be smaller during third trimester of fetal life but were normal size as infants. By contrast, children with increased aortic root diameter or left ventricular mass tend to be larger during fetal life, but of similar size during infancy (Table 3) (54). School age children with clustering of CVD risk factors had a smaller first-trimester fetal crown-rump length, lower second and third trimester estimated fetal weight but more rapid growth from 6 months onward. This suggests that even first-trimester fetal growth relates to subsequent cardiovascular risks (71). As adults, women rather than men with LBW (≤2.5 kg) have higher fasting plasma glucose, insulin, diabetes, and metabolic syndrome (Table 3). In both genders, height increased with BW, whereas BMI and waist circumference have a U-shaped association with BW (58).

Adequate nutrition and micronutrient density such as iron, copper, zinc, iodine, selenium, and vitamin A and D are prerequisite for fetal growth. In industrialized countries, food containing essential micronutrients is likely to be more expensive decreasing the dietary quality in low-income groups (72, 73). Even in countries without absolute food shortage (e.g., Sri Lanka) 25% of mothers and 33% of all children were malnourished and at risk of anemia, mental illness, or poor immune system (74). Exposure to unexpected contraction in the first trimester was associated with a decrease in BW which was stronger for women at home with <12 years’ education and associated with increased risks for FGR (OR 1.5; 95% CI 1.1–2) (75). In recognition of the important impact of maternal nutrition and the crucial importance of preventive care, reduction of poverty and hunger during pregnancy is a defined millennium goal of the United Nations.

#### Parity

Older studies have demonstrated that multiparity independently increases maternal CVD risks in later life (31) (Table 2). A “J” shaped association between number of children and CVD was observed, lowest among those with two children and increasing with each additional child beyond two by 30–47% for women and by 12% for men (32) (Table 2). Specifically, in women, the number of children was inversely correlated with high-density lipoprotein (HDL) cholesterol and positively associated with TGs and diabetes. It has been concluded that lifestyle risk factors combined with child rearing result in obesity and increased rates of CVD. The 9% increase in CVD risk in women with only one child has been attributed to coexisting fertility conditions such as polycystic ovary (PCO) (76).

#### Hypertensive Disorders of Pregnancy (HDP), PE

Risk factors for HDP can be broadly categorized as *personal, cardiovascular, metabolic*, and *prothrombotic* (77). Approximately 80% of women with a history of PE have at least one risk factor of which the cardiovascular risk profile is most prevalent, followed by hyperhomocysteinemia, metabolic syndrome, and thrombophilia (78, 79). Both circulatory and metabolic risk profiles were associated with earlier onset PE; FGR was more likely in patients with a diastolic BP above 80 mmHg (80). All first-trimester prediction algorithms for PE identify surrogate markers of cardiovascular and metabolic health as independent contributors (77). The most recent and largest systematic review and meta-analysis on risk factors for PE in relation to clinical risk factors included more than 25 million pregnancies (81): women with antiphospholipid antibody syndrome had the highest rate of PE (17.3%, 95% CI 6.8–31.4%). Those with prior PE had the greatest relative risk (RR) of 8.4 (7.1–9.9). Chronic hypertension ranked second with a RR of 5.1 (4.0–6.5), pre-gestational diabetes had a RR of 3.7 (3.1–4.3), pre-pregnancy BMI > 30, and ART were other risk factors suggesting that the presence of any one might suffice to designate a woman as “high risk” and support clinical prediction for the use of early prevention (81). The parallel rise in PE and maternal long-term complications supports the concept that early-pregnancy risk profiles are causally linked to maternal long-term health. Accordingly, the American Heart Association guidelines on female CVD include GDM and HDP in their risk assessment (82). Although balanced diets and active lifestyles reduce the risk for DM in women with GDM, adoption of these health behaviors is low (83).

Placental invasion, size, and function are sensitive to maternal blood flow disturbances and the placenta modulates fetal responses to the environment. The “gateway” to the fetus modifies epigenetic marks and placental gene expression leading to gender-specific diseases (84). The placental lesions of PE and severe FGR, such as “atherosis in the placental bed,” are similar to atherosclerosis suggesting common genotypes, phenotypes, and earlier mortality (85). Mothers with PE have atherosclerotic disease such as angina pectoris, cardiac insufficiency, or renal disease within a mean interval of 11 years and earlier mortality is evident by 20 years after delivery in these women (86, 87).

Maternal placental syndromes combined with FGR increase the downstream risk for early hospitalization for heart failure, cardiac dysrhythmia, and CVD follows this pattern (34, 35) (Table 2) suggesting that dysfunction in both placental compartments amplifies the risks. Affected women should therefore have their blood pressure and weight assessed by 6 months postpartum, and a healthy lifestyle should be emphasized. Following coronary artery revascularization, middle-aged women are at higher risk for death than men. After a mean of 5 years, 41 deaths (2.2 per 100 person years) occurred in women compared to 1.1 in women without maternal placental syndrome (HR 1.96; 95% CI 1.29–2.99). The risk of death was significant in women with placental abruption (HR 2.79; 95% CI 1.31–5.96), placental infarction (HR 3.09; 95% CI 1.23–7.74), and PE (HR 1.61; 95% CI 1.00–2.58). Women with placental syndrome in two pregnancies had the highest HR of death of 4.31 (95% CI 1.71–10.89). This should be included in the informed consent process (33).

Children born to pre-eclamptic mothers are at increased risk for high BP, stroke, cognitive delay, and depression (Table 3) (55). As young adults, these children have a 2.5-fold increased risk of a QRISK Score above the 75th centile (95% CI 1.32–4.56, *p* = 0.004). Consideration of additional factors would allow identification of a cohort with hypertension (Table 3) (56). PE leads to a 40% elevated risk of later serious CVD (88); 30% of all 20-year old’s with high BP had mothers with PE (95% CI 1.3–7.0; *p* = 0.01) (56). The recognition of these associations by family practitioners and pediatricians raises the possibility of tailored interventions to prevent adult hypertensive disease. The discussion on the optimal management approach to HDP during pregnancy is ongoing (89, 90).

First-trimester pregnancy-associated plasma protein-A was one of the first serum biomarkers noted to correlate with placental function and fetal growth (91). Now, more complex first-trimester screening algorithms for PE and FGR offer individual risk prediction with up to 90 and 60% sensitivity, respectively (5, 92, 93); 91% of women that are test positive at the first-trimester screen have cardiovascular and metabolic conditions amenable to therapy (8). Since many of these risk profiles pre-date before or persist after pregnancy it appears that first-trimester screening for placental disease is not only cost-effective but could have therapeutic benefits that reach far beyond pregnancy (94).

#### Prematurity

Prematurity and low BW are endpoints of several potentially different etiologies (95). Women who had a preterm birth more frequently develop CVD and type 2 diabetes (4, 36). There is a negative correlation with gestational age at delivery and the rates of later maternal diseases. In a recent systematic review, SPB increased maternal risks of developing or dying from IHD, stroke, and overall CVD (28, 96) (Table 2).

Following preterm birth, the offspring is also at higher risk for elevated BP levels in adulthood and insulin resistance in infancy (97). It is unknown if this is attributable to interactions between peripartum exposure to inflammatory cytokines, cardiovascular effects of pulmonary dysmaturity, and placental dysfunction (98–100).

#### Stress

Acute stress responses activate the hypothalamus–pituitary–adrenal axis (HPA) and the immune system to enable the organism for environmental threats. However, prolonged activation of the stress response may have adverse consequences. Maternal stress, anxiety, and psychological maternal disease can evoke immediate changes in blood flow to the uterus, fetal heart rate, or fetal movements (FM). However, they also induce long-term changes in fetal growth, metabolism, behavior, and cognition. Since there are no direct neural connections between mother and fetus, acute and chronic responses are likely elicited by neuroendocrine, autonomic, or vasodilatory input. Low BW by itself is associated with “hostility” in adult life, e.g., a rival cynic personality with mistrust and negative affections, which again is combined with CVD (57) (Table 3).

It is fascinating to imagine that the fetus may actively contribute to its own epigenesis as FM between 20 and 38 weeks transiently stimulate maternal sympathetic arousal prepare women for nurturing without becoming desensitized (101). Listening to music and singing lullabies has been shown to reduce women’s experience of stress, anxiety, and depression and might simultaneously stimulate and be remembered by the fetus (102, 103).

The interplay of maternal stress on metabolic disorders in the next generation has been investigated: only 2/45 known type 2 diabetes susceptibility genes are associated with LBW, indicating that the association is mainly non-genetic. The developing fetal brain requires some, but not overwhelming stress. FGR is associated with poor school education, smoking, drinking habits, poor social activities of mothers, and poor maternal social support (59). Even exposure to modern media “attacks” seems to reduce BW by 50 g (104). Maternal exposure to the death of a close relative is also correlated to LBW, where deregulation of the HPA was most marked during the second trimester when spiral arteries invade trophoblastic cells (105). Endocrine factors, such as β-HCG or progesterone, play gender-specific roles for growth and disease. In a multivariable regression model, increase in maternal progesterone by 1 ng/ml during the first trimester increased girls’ BW by 10.2 g (95% CI 2.03–18.31); perceived worries (and smoking) predicted FGR in boys irrespective of progesterone levels (106). Fascinating reviews on maternal stress have been published (67, 107).

#### Smoking, Toxic Agents

Maternal smoking is one of the commonest modifiable risk factors. It has been estimated that active or passive smoking during pregnancy is responsible for at least 20% of infants with LBW (108). The effect is dose dependent (adjusted OR 2.40 for 0–9, 2.68 for 10–15, 2.88 for >15 cigarettes daily). Parental smoking also increases CVD risk in the offspring (109). After puberty, the effect of parental smoking was positively associated with BMI (*p* < 0.001) with a significant dose response for each additional 10 cigarettes. The associations of maternal smoking were stronger than for paternal smoking. At age 32, offspring of at least one smoking parent had higher BMI and waist circumference. Adjusted weight at age 17 was 63.2 kg for offspring of non-smoking parents compared to 64.6 kg of at least one smoking parent and at age 32, 68.3 and 70.5 kg, respectively (60) (Table 3). In many countries, pregnant mothers are exposed to wood fuel smoke. The carbon monoxide depresses placental energy-dependent processes and amino-acid transport and exhibits a reduction of infants’ adjusted mean BW by −186 g (110). Variation in genes encoding enzymes modify the associations between maternal smoking and BW, but the contribution of epigenetic mechanisms, rather than genetic, underlie the long-term effects of smoke exposure as shown in aberrant placental metabolism, syncytial knot formation, or markers of placental oxidative damage (111).

### Health Risks Predominantly Attributable to a Metabolic Risk Profile

Between 2011 and 2012, an estimated 14.4% of US women over 20 had high total cholesterol; overall, 65% of US women are overweight or obese, with highest rates among non-Hispanic black women (14). The International Diabetes Foundation defined metabolic syndrome as BMI > 30 kg/m^2^ or WC > 80 cm (women), and at least two of the following criteria: fasting glucose > 5.6 mmol/l (100 mg/dl) or diabetes, cholesterol > 1.3 mmol/l (50 mg/dl), or medication use to low HDLs, TG levels of >1.7 mmol/l (150 mg/dl) or specific treatment and a BP > 130/85 or use of antihypertensive medication (112). It remains unclear, in how far single criteria are associated with a higher risk for stroke or CVD and the precise scientific concept of the metabolic syndrome remains controversial. The WHO has put forward specific diagnostic criteria relating to BMI, blood pressure, proteinuria, TG, and HDL (113). Each component of the metabolic syndrome increases risks for PE especially when C-reactive protein is elevated (114). *Vice versa*, women that develop PE exhibit more pronounced insulin resistance and dyslipidemia, which frequently continues after pregnancy (115–118).

The rise in the prevalence of childhood and adult obesity in low- and high-resourced countries led WHO’s Director General to establish a Commission on Ending Childhood Obesity, which stressed the need for concerted and sustained action, early in the life course of mothers (and fathers) (119). The UN General Assembly proclaimed 2016 a Decade of Action on Nutrition calling upon governments to address the diet-related burden of disease (120). A series on preconception and maternal obesity in The Lancet Diabetes & Endocrinology 2016 and 2017 suggests new directions that such an initiative could take (121) and demonstrates how uncontrolled GDM is responsible for a transgenerational passage of obesity.

#### (No) Breastfeeding

Several studies have shown that breastfeeding is associated with decreased activation of the HPA axis, a blunted BP response to stress, and increased fat mobilization (122–124). In a prospective Danish cohort, an inverse association was observed for breastfeeding duration and weight retention up to 18 months correlating with anthropometric measures 7 years after delivery (37) (Table 2). In mothers, who already have symptoms of GDM, breastfeeding is protective against type 2 diabetes.

Infant feeding characteristics are associated with subsequent risk for maternal DM within 2 years (38) (Table 2). Potential mechanisms for these associations include prolactin-mediated preservation of pancreatic β-cells, less inflammation, and improved endothelial function. To estimate the effect of breastfeeding on maternal health, mice were randomly divided into lactated (L) and non-lactated (NL) animals (120). At 9 months, the NL group weighed significantly more compared with the L group had significantly higher systolic BP, lower ejection fraction, and higher renal artery resistive indices compared with L mice which all suggested that lactation has a direct beneficial effect. Large systematic reviews covering >9,000 abstracts and approximately 400 individual studies have demonstrated that breastfeeding reduces risks for type 2 diabetes, breast, and ovarian cancer. Early cessation or not breastfeeding was associated with a higher risk of postpartum depression. There was no relationship between a history of lactation and the risk of osteoporosis (125). Lactation duration was found to be inversely associated with common carotid intima-media thickness at 20 years; mean differences between a duration of ≥10 months compared with 0 to <1 month ranged from −0.062 mm for unadjusted models to −0.029 mm if adjusted for pre-pregnancy BMI, cardiometabolic risk factors, parity, smoking, and sociodemographics (*p* trend: 0.01) (38). It is not yet clear whether all observed associations between breastfeeding and maternal health are causal since breastfeeding women have a healthier lifestyle overall. Nevertheless, it was estimated that low breastfeeding rates in the US result in 4,981 excess cases of breast cancer, 53,847 cases of hypertension, and 13,946 cases of myocardial infarction. Suboptimal breastfeeding is therefore supposed to incur a total of $17.4 billion in cost to society/year resulting from premature death (95% CI $4.38 to 24.68 billion), $733.7 million in direct costs (95% CI $612.9 to 859.7 million), and $126.1 million indirect morbidity costs (95% CI $99.00 to 153.22 million). There was a non-significant difference in additional premature deaths before 70 years (126).

Infants benefit from breastfeeding due to protection from infections and biologic signals for promoting cellular growth and differentiation. Breastfeeding reduced the severity of respiratory problems in the first 27 weeks of life (RR 0.70; 95% CI 0.55–0.88) (127) and reduces risks for acute otitis media, non-specific gastroenteritis, lower respiratory tract infections, atopic dermatitis, asthma, obesity, type 1 and 2 diabetes, childhood leukemia, sudden infant death syndrome, and necrotizing enterocolitis (125). The American Academy of Pediatrics recommends exclusive breastfeeding for approximately 6 months, followed by continued breastfeeding with complementary foods for 1 year or longer (128). The American College of Obstetrics and Gynecology asks for a multidisciplinary approach involving practitioners, family members, and child care providers to support breastfeeding mainly for underserved women (129). Breastfeeding promotion is a practical, low-cost intervention to prevent CVD, obesity, and diabetes in high-risk women, with the potential for benefits that are complementary to lifestyle interventions targeting weight loss.

#### Pre-Pregnancy Obesity and Excessive Weight Gain during Pregnancy

Overweight (BMI > 25 kg/m^2^) and obesity (BMI > 30 kg/m^2^) have become global risk factors for NCDs (130). Obesity before and during pregnancy leads to an increase of maternal mortality; more than 50% of all maternal deaths in Great Britain were overweight or obese (131). The accumulation of visceral fat tissue correlates with increasing insulin resistance and a metabolic syndrome (132). Pregnancy itself leads to obesity: approximately 75% of women are heavier 1-year postpartum than they were pre-pregnancy (133). Increased abdominal fat mass (134), which also characterizes aging (135), may affect long-term maternal health because abdominal fat mass predicts mortality better than weight or BMI (136). This is also the reason why a body fat index—a novel ultrasound index evaluating central maternal fat—seems to be more informative than BMI in terms of prediction of obstetric complications particularly for subsequent development of GDM. Also women with normal pre-pregnancy BMI need to be informed about the recommendations by the Institute of Medicine. Excess weight gain increases fat mass, especially in women with preconception obesity (137, 138). Excess pregnancy weight gain increases the risk for lifelong visceral fat retention (39, 137, 139, 140) (Table 2).

Mothers with excessive weight gain are less likely to breastfeed (141). In a cohort of 46,688 women, a BMI ≥ 30 kg/m^2^ was associated with higher adjusted rates of cardiovascular events and related hospitalizations (142). Truncal obesity as defined by WC or waist/hip ratio has been shown to be more strongly related to certain cancer types than obesity as defined by BMI (143). Possible mechanisms that relate obesity to cancer risk include insulin resistance and chronic hyperinsulinemia, increased production of insulin-like growth factors, or high bioavailability of steroid hormones because adipose tissue-derived hormones and cytokines (adipokines), such as leptin, adiponectin, and inflammatory markers, may reflect mechanisms linked to tumor genesis.

Maternal obesity or pre-gestational DM is associated with fetal myocardial functional changes as early as the first trimester, which could explain the predisposition of offspring to CVD later in life (29, 41). Offspring of mothers with excessive weight gain and a BMI > 30 kg/m^2^ are more frequently obese at age 16 compared to offspring of mothers with normal weight gain, even after adjusting for age, socioeconomic status, sex, or BW (144). As adults they demonstrate reduced life quality and life span (HR: 1.35; 95% CI 1.17–1.55) (145). These Scandinavian epidemiologic data match with animal experiments demonstrating that rats on a high-calorie diet before and during pregnancy demonstrate adipogenesis and are “programmed to early death, e.g., a shorter life” (146). Endothelial lesions, increased number of fat lobule *in utero*, as well as the U-shaped correlation of BW and obesity might play a role (3). Additionally, exposure to obesity and high-fat diet before, during, and after pregnancy promote appetite over satiety neurons in the hypothalamic arcuate nucleus leading to offspring hyperphagia and obesity (147). Finally, a high-caloric or high-fat maternal diet modulates the fetal gut microbiome and gut–brain axis causing a persistent predisposition to metabolic disease and obesity in the offspring (148, 149).

Maternal obesity is associated with sex-specific differences not only in fetal size but also in neurodevelopment reflected by gene expression signatures and the brain transcriptome. Especially male embryos of dams on the high-fat diet had a significantly lower BW than controls; dietary change in pregnancy resulted in significantly more dysregulated genes and pathways in male than in female brains (386 vs. 66, *p* < 0.001) (150).

#### GDM and Pre-Existing Diabetes during Pregnancy

Type 1 or type 2 diabetes may exist before pregnancy; GDM is defined as glucose intolerance first diagnosed in pregnancy and is associated with subsequent hyperinsulinemia, dyslipidemia, type 2 DM, hypertension, and CVD (151). Already in the 1950s, Pedersen et al. reported the association of GDM with DM and fetal macrosomia [c.f. (152)]. In the 1980s, Freinkel described the impact of GDM on fetal long-term health and together with Metzger on maternal glucose tolerance after birth; 30% of women with GDM have a persisting glucose tolerance and develop type 2 diabetes within 10 years (153–155). Worldwide, the incidence of GDM has increased to 7–14% (156). Weight gain above the IOM norms and GDM are associated: a case–control study of 800 women with excessive weight gain but normal glucose tolerance showed a 50% increase of GDM compared to controls with normal weight gain (118).

The hypothesis that pre-existing risk profiles play a role is supported by the fact that first-trimester prediction of GDM by history or biochemical–biophysical tests has sensitivities as high as 80% (7). The risk to develop overt DM increases with maternal age and accelerated almost 10-fold for women with GDM resulting in cumulative 15-year risk of 25% (156). A comprehensive systematic review including 205 relevant reports, 20 studies, 675,455 women, and 10,859 type 2 DM events confirmed the increase of type 2 DM compared to normoglcyemic women (40) (Table 2).

Fetal programming related to GDM and DM is complex since both, low and high BW are associated with the development of a metabolic risk profile in later life. A significant association between low (OR 2.15, 95% CI 1.29–3.50) and high BW (OR 1.97, 95% CI 1.12–3.45) and later development of GDM was shown with a U-shaped relation between BW and risk of GDM (61). In families or regions where GDM was not known before an “epigenetic mismatch” between prenatal and postnatal nutrition plays an increasing role.

The hypothesis “fetal origins of disease” was proposed to explain associations between low BW and impaired glucose tolerance or CVD (157–161). The thrifty phenotype hypothesis suggests that early malnutrition induces poor development of pancreatic β-cell mass and programs the metabolic syndrome (157–162). LBW was related to high concentrations of split proinsulin, a sign of beta-cell dysfunction, linked to later high blood pressure (163, 164) and to metabolic abnormalities in combination with low physical activity and/or high-energy intake (165). The British Maternal Nutrition Study correlated prenatal micronutrient deficiency with increased insulin resistance in childhood: the offspring of mothers with combined high folate and low vitamin B12 levels were insulin resistant (166). Similarly, prenatal famine exposure was associated with impaired glucose tolerance and insulin secretion in adulthood (167, 168). Before, it was already demonstrated that a pregnancy with a LBW child indicates a risk for significantly lower rates of insulin, C-peptide, and proinsulin responses than controls. Insulin sensitivity was increased in the FGR compared to the control group (169).

Children of mothers with GDM and fetal macrosomia are at higher risk of childhood obesity and its consequences (170). Metabolic markers such as insulin resistance and high TGs are present in 21% before puberty (171) and at age 11, maternal GDM with fetal macrosomia increased the risk of metabolic syndrome 3.6-fold over controls with isolated macrosomia (63). Similarly, as adults these children are at higher risk for DM and metabolic syndrome (171, 172). A large Danish study of women with DM confirmed that as adults the offspring was at increased risk for CVD (adjusted OR 1.46; 95% CI 1.16–1.83) and for insulin-dependent DM (adjusted OR 4.7; 95% CI 3.9–5.8) compared to offspring of non-diabetic mothers. CVD was associated with FGR rather than macrosomia (OR 1.29; 95% CI 1.24–1.35) (173). Accordingly, undernutrion during pregnancy was linked to decreased glucose tolerance up to age 58 (62) (Table 2).

#### Cesarean Rates and Subfertility

Cesarean delivery and later childhood obesity are associated independently of the fact that Cesarean rates are *per se* higher in obese women and differences in the infant intestinal microbiome has been postulated as a potential explanation (174). Subfertility such as premature ovarian insufficiency or PCO is another factor that increases risk for CVD and metabolic disease (42, 76).

Children conceived through artificial reproduction techniques have higher glucose levels compared to controls (64). At the age of 5–6 years, glucose levels were increased by 0.4 mmol/l (95% CI 0.2–0.6) and 0.2 mmol/l (95% CI 0.0–0.5), respectively. Similarly, systolic and diastolic BP was elevated by 0.8 mmHg (95% CI −0.2 to 1.8) and 1.4 mmHg (95% CI 0.6–2.3). Since the duration of infertility correlates with BP in the offspring and with PE in the pregnant women, epigenetic and genetic factors have been implied (64).

### Predominantly Thrombotic Risk Profile: Thrombophilia, Systemic Lupus Erythematosus (SLE), and Antiphospholipid Syndrome (APS)

Most women with a thrombotic risk profile are already aware of their diseases before pregnancy.

But pregnancy is regarded as a prothrombotic state due to its impact on coagulation. It modifies the disease or makes it visible when no symptoms were known before.

Coagulation disorders such as thrombophilia, SLE, and APS are recognized risk factors for placental dysfunction, FGR, and PE. In these conditions, aspirin or heparin therapy may decrease the rate of thrombosis and possibly of placental disease and adverse outcome (175, 176). The generalized administration of prophylactic anticoagulants is not supported until RCTs show a benefit (175). Patients with APS, SLE, and triple antiphospholipid antibodies or prior thrombosis are at risk for FGR or recurrent thrombosis and seem to have a better fetal outcome when treated with anticoagulants (177). Women with thrombophilia, SLE, and/or obstetric APLS during pregnancy have lifelong thrombosis risks requiring long-term anticoagulation and awareness during risk situations; patients with arterial events should be treated aggressively.

During pregnancy, low-dose aspirin is likely to address underlying factors that promote a prothrombotic risk profile. However, therapy of hyperhomocysteinemia may also be worthwhile as high first-trimester homocysteine levels increase the risk of PE threefold to fourfold (178, 179).

Low folate intake is an important contributor to increased homocysteine levels and is significantly more common in women who develop PE (180). Modification of homocysteine levels requires high-dose folate and an RCT evaluating a 4 mg folate daily is ongoing (181, 182). It remains to be determined if folate will benefit all women or specifically those with elevated homocysteine levels.

Children of mothers with a prothrombotic risk profile have only been investigated in small series: the neurodevelopment of 30 children born to mothers with SLE and/or APS with IgG antibeta2-glycoprotein I positive for the same antibodies at birth have been examined up to 9 years postnatally according to a Child Behavior Check List, rheumatologists, and pediatric characteristics. In all children, neurological physical exam and intelligence levels were normal. Mild behavior disorders were shown in three children possibly related to maternal disease or prematurity (183).

## Discussion

**Figure 2:**
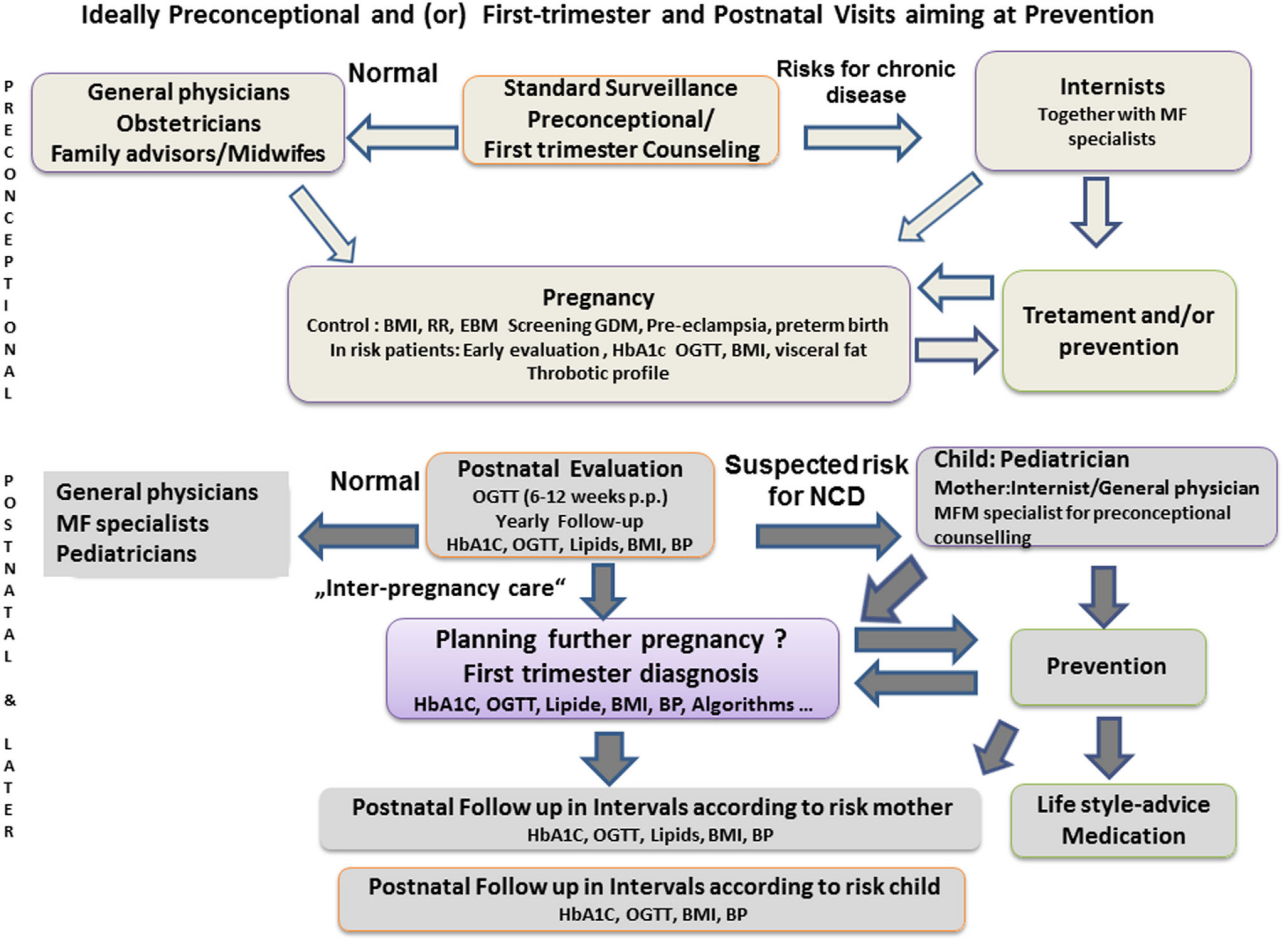
**Schematic proposal for health-care concepts to intensify interdisciplinary cooperation, pre-conceptional counseling, and postpartum consultation of women with pregnancy complications, according to Carson (112) or Bohrer and Ehrenthal (36)**. BMI, body mass index; BP, blood pressure; EBM, evidence-based medicine; GDM, gestational diabetes mellitus; MF, maternal fetal; NCD, non-communicable disease.

In 2011, the General Secretary Ban Ki-Moon declared during the first NCD-conference of WHO and the United Nations: “We strive for an international commitment that puts NCDs high on the development agenda.” Confirming a match of epidemiological data with molecular mechanisms described in human or animal research during pregnancy, opens opportunities to couple the prevention of NCDs with reduced health-care costs. This is particularly relevant for maternal-infant care (Figure 2). There are periods in our life cycle in which we are particularly susceptible to epigenetic influences; mediated by mechanisms that include DNA methylation, histone modification, and RNA silencing. It appears that the pathophysiology of adverse pregnancy outcome can have its origins during fertilization, gametogenesis, embryonic, fetal, and placental development and translates into downstream long-term health impacts (184, 185). We have shown that many pregnancy complications are associated with maternal and infant health risks in later life. By recognizing the sentinel circumstances during key periods in pregnancy, we are opening a unique window of opportunity for interventions to improve maternal-child health. To increase our knowledge and relevant consequences for long-term health, we have to associate findings from animal experiments and clinical measurements with transgenerational data from large populations.

New evidence is leading us to revise our understanding on the origins of placental disease and its interdependence with maternal and child health (19). It has been presumed that the increased uterine artery resistance and abnormal physiologic transformation of the placental vasculature is the initiator of placenta-based diseases such as PE and FGR. However, observations in other maternal vascular beds that are independent of early trophoblast function and the documentation of maternal risk profiles long before placental disease has developed, challenge these concepts (19, 77, 185, 186).

Even in normal pregnancy, myocardial and ventricular function decline after the second trimester (187). The findings of a drop in stroke volume index, impaired myocardial relaxation with diastolic dysfunction, and eccentric remodeling at term are suggestive of cardiovascular maladaptation to the volume overload in some apparently normal pregnancies. Following pregnancies with FGR and especially PE has shown a low cardiac output, high resistance circulatory state, asymptomatic global diastolic dysfunction, and poor cardiac reserve (188). Postpartum follow-up has demonstrated remodeling in patients with PE, but asymptomatic left ventricular dysfunction and hypertrophy were significantly higher in preterm PE compared with term PE or matched controls. The risk of developing essential hypertension within 2 years was higher in both women with preterm PE and those with persistent left ventricular abnormal function or geometry. Specifically, these women are more likely to experience recurrent placental disease (189). Accordingly, pre-existing risk constellations seem to interact with placental development in women that develop PE or placenta-based FGR and that their risks are increased by the adverse pregnancy outcome. The synergy between individual risk constellation and their amplification by pregnancy complications is likely to be responsible for the downstream health effects as described above (190). To determine the thresholds where disease occurs requires large perinatal registers that are initiated before conception and follow women and their offspring.

The question whether later diseases in maternal life are caused by the disease itself or a pre-existing condition can also be illustrated by perinatal registers as it was performed for end-stage renal disease (ESRD). It could be concluded that familial aggregation does not explain increased ESRD risk after PE, but that PE *per se* leads to kidney damage (191).

Gestational hypertension and PE share baseline risk factors, such as a family history of DM, of myocardial infarction before 60 years and elevated TG levels while physical activity is protective (192). Extending registries across generations will amplify our ability to influence public health by even earlier preventive care to benefit mothers, fathers, and their children (193). The effects of FGR and PE on survival rates of fetuses and mothers drawn from the Norwegian registry mirror both genetic and epigenetic influences and match with the topic of this paper (Table 4).

**Table 4:** **Perinatal death rates as relative risks (RRs) and later maternal death rates form cardiovascular disease in later life correlated with gestational age at birth and pregnancy disease [preeclampsia (PE) and fetal growth restriction], designed from recent data of the Norwegian data base, personal communication and with gratitude to Rolv Skjaerven, 2017**.

PE during first pregnancy	Gestational weeks at delivery	Birth weight (*Z*-score)	Perinatal death (RR)	Maternal death (HR)
Yes	≥37	Large (>0)	2.0	1.8
Yes	≥37	Small (<0)	6.3	1.5
Yes	35–36	Large	4.1	**5.2**
Yes	35–36	Small	23.8	0.9
Yes	≤34	Large	29.3	**11.3**
Yes	≤34	Small	**79.4**	2.3
No	≥37	Large	1 (reference)	2 (reference)
No	≥37	Small	2.9	1.3
No	35–36	Large	4.3	2
No	35–36	Small	21.3	2.5
No	≤34	Large	51	2
No	≤34	Small	**131**	2.8

**Figure 3:**
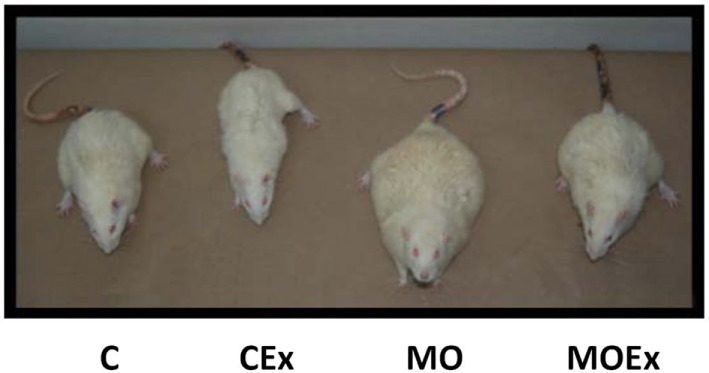
**Representative pictures of male offspring of rats at postnatal day 650**. C, control diet; CEx, control diet + maternal exercise intervention; MO, maternal obesity; and MOEx, maternal obesity + maternal exercise intervention, with gratitude, according to Nathanielsz et al. (146).

Transgenerational research in rats from Nathanielsz et al. (146) not only show how obese mothers transfer obesity to their children, but also demonstrate that the effect can be limited by physical activity or a diet during pregnancy. The pictures of the second generation not only help to understand the epigenetic pathophysiology but can motivate patients with a high BMI to stick to IOM guidelines and to be physically active (Figure 3).

Continuous family care is relevant with a focus on selected time periods:

(a) Prenatal and early postnatal life offers a window of epigenetic plasticity when environmental factors may condition the body in ways that shape disease risk in later life (194, 195).
(b) The past as experienced by siblings, parents, grandparents, and possibly earlier generations becomes relevant for understanding our disease risks today and tomorrow (193).

In Scandinavia, registry-based perinatal epidemiology has shown the importance of sibling and generation data which allow linking of birth records across generations (193). These population-based linked materials provide research opportunities beyond cross-sectional studies where observations are often based on studies of the woman’s first pregnancy. But women who stop reproducing after one pregnancy have different mortality rates than women with two or more pregnancies (196). Studying the next pregnancy conditional on outcomes of previous pregnancies is challenging and shows risk heterogeneity between women (193). Intergenerational data reveal the influence of socioeconomic and behavioral factors, and not only genetic inheritance (197).

Compared with the task of family doctors of previous times, obstetricians, and MFM specialists are predominantly focused on prenatal care and obstetric emergencies with less emphasis on the long-term outcome of women and their families. In most countries, there is not even a continuity of care between obstetric providers and other care specialties. As a result, research, development, and clinical care across this critical health-care frontier are disproportionally sparse.

As MFM specialists are in the advantageous position to screen, diagnose, and manage pregnancy-related complication in an index pregnancy they are ideally positioned to initiate care paths after pregnancy. Sentinel risk profiles need to be incorporated into care models, which will allow the initiation of personalized care paths for mothers and infants (8, 198). In the US, the diabetes prevention trial already aims to introduce a balanced diet and more active lifestyle to reduce later risks for diabetes (199). Models exist to use BP, lipids, visceral fat, BMI, and glucose tolerance at 6 and 12 months after high-risk pregnancies to define the need for inter-pregnancy care, lifestyle interventions, or therapy within specialized clinics (200–204). The fact that pregnant women are more sensitive for health-care advices should be used as a chance to intervene as early as possible (36). A potentially useful tool would be to modify the maternal “passport” as it is currently utilized in many European countries to produce a lifelong health record as required. Widely available computer technologies and app’s can be designed for targeted information about risks, interventions, and evidence-based concepts and are preferred to booklets in the younger generation (205).

Meanwhile, a first guideline for follow-up of patients after previous PE has appeared, whereby optimization of modifiable cardiovascular risk factors is recommended for reproductive and pregnancy-related disorders to reduce the risk of future CVD (206). Progress will eventually become inevitable as focused patient history taking, pregnancy risk algorithms, and the existing obstetric care platform already meet WHO criteria for screening tests (207). It is our responsibility to incorporate interdisciplinary care algorithms (87, 208) (Figure 2).

We need to realize how epigenetic findings relate to questions of social and environmental justice and not only to individual responsibility (24). This requires recognition of the presented associations as an opportunity to modify preventive and long-term care. Such progress hinges on widespread patient and health-care provider education about the unique opportunity to identify and treat modifiable risk factors for adverse health outcomes (209, 210). Strategies to reduce long-term and intergenerational risks associated with pregnancy disorders should include access to interdisciplinary teams to substantially affect future pregnancy outcomes and chronic illness.

Policy makers need to establish preventive interventions and need to better tackle long-term risks and inequalities of perinatal care. The future MFM specialist will be less invasive, give less medicine but will interest patients in the cause and prevention of disease as Thomas Edison stated (151). We have to reduce health illiteracy, the misbalance between responsible and irresponsible resource management and thereby the burden of increasing rates of chronic diseases (211).

## Author Contributions

BA and AB had a substantial contribution to the conception and design of the work and its interpretation. BA had drafted the work and both BA and AB have revised it critically. Final approval and agreement to be accountable for all aspects are agreed on by BA and AB.

## Conflict of Interest Statement

This manuscript was written in the absence of any commercial or financial relationships that could be construed as a potential conflict of interest. The reviewer, AF, declared a shared affiliation, though no other collaboration, with one of the authors, AB, to the handling editor, who ensured that the process nevertheless met the standards of a fair and objective review.
